# Small Sample Machine Learning for Predicting Composition Changes in Chemically Pretreated Kapok Fiber

**DOI:** 10.1021/acsomega.6c04765

**Published:** 2026-09-23

**Authors:** Yitong Niu, Ireland LaBass, Ying Ying Tye, Tristan Smith, Sicheng Wang, Yunxiang Li, Ting Han, Cheu Peng Leh

**Affiliations:** † School of Industrial Technology, 26689 Universiti Sains Malaysia, 11800 Gelugor, Penang, Malaysia; ‡ Division of Chemistry and Biochemistry, Texas Woman’s University, Denton, Texas 76204, United States

## Abstract

Kapok is a lightweight hollow tropical fiber with potential as a renewable feedstock, but its waxy surface and lignin-containing matrix complicate aqueous processing. This study evaluated whether limited-data machine learning could predict kapok fiber composition after aqueous chemical pretreatment using only three process descriptors. Kapok fibers were pretreated using dilute H_2_SO_4_, hydrothermal water, and NaOH across a shared window of residence time, temperature and pH-associated pretreatment environment. A data set of 33 pretreatment runs was constructed, with cellulose, hemicellulose and lignin contents used as composition targets after correction with solid yield. Single-output and wrapper-based multitarget regression models were screened and then evaluated using leave-one-out cross-validation to reduce dependence on a single train–test split. The LOOCV results showed target-dependent predictability. In single-output modeling, cellulose was the most predictable component, with GBDT giving the strongest performance (*R* ^2^ = 0.782; RMSE = 4.185 percentage points), whereas hemicellulose and lignin showed weaker prediction. In wrapper-based multitarget modeling, GBDT improved simultaneous prediction of cellulose, hemicellulose and lignin, giving *R* ^2^ values of 0.909, 0.653, and 0.672, respectively. Feature-importance analysis indicated that temperature was the dominant descriptor for polysaccharide-related composition changes, whereas the pH-associated pretreatment environment contributed more strongly to lignin-related variation. These findings position limited-data machine learning as a useful exploratory tool for modeling pretreatment–composition relationships in kapok fiber, while also highlighting the need for cautious validation when sample size is small.

## Graphical abstract

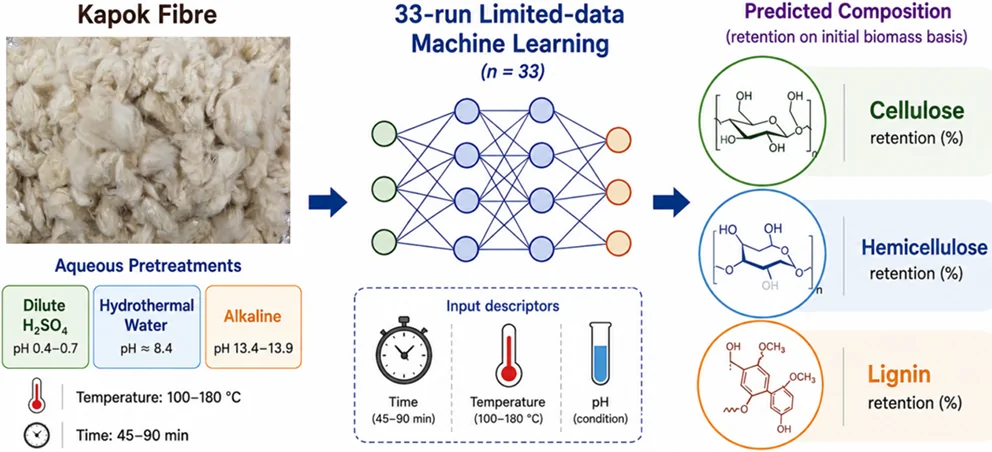

## 1 Introduction

Lignocellulosic fibers are gaining increasing attention as renewable feedstocks for biobased chemicals, fuels, and functional materials, yet their conversion efficiency is limited by the native arrangement of cellulose, hemicellulose, and lignin in the plant cell wall.^1,2^ Among nonwood tropical fibers, kapok is distinctive for its hollow lumen, low density, and wax-rich cuticle, attributes that enable applications such as absorbents and insulation but also amplify mass-transfer limitations and enzymatic inaccessibility.^3^ A hydrophobic surface layer, comparatively high lignin fraction, and lumen collapse upon drying render untreated kapok highly recalcitrant to aqueous processing.^4,5^ Consequently, effective valorization requires pretreatments that disrupt or redistribute the lignin–hemicellulose matrix while preserving sufficient cellulose for downstream use.^6,7^ A clearer understanding of how different pretreatment chemistries reshape kapok’s composition is therefore essential for rational process design.

Dilute acid, hydrothermal autohydrolysis, and alkaline pretreatment constitute three widely adopted aqueous approaches for reducing lignocellulosic recalcitrance.^8^ Dilute acid conditions catalyze hemicellulose solubilization and generate a cellulose-rich solid with limited lignin removal.^9^ Hydrothermal pretreatment removes hemicellulose through autohydrolytic reactions and partially opens the fiber structure in water-based pretreatment systems, with lignin typically redistributed rather than solubilized.^10^ Alkaline pretreatment creates a contrasting environment in which lignin and lignin–carbohydrate linkages are cleaved, promoting delignification and fiber swelling and enhancing cellulose accessibility at elevated severity.^11–13^ Studies on kapok and related fibers have documented structural and digestibility changes under these regimes, yet systematic, condition-resolved comparisons across acid, hydrothermal, and alkaline spaces remain limited.^14–16^

Much of the available evidence is fragmented across studies that examine only narrow regions of time–temperature space or treat pH in a simplified manner, making it difficult to generalize compositional trends across pretreatment types.^17^ Severity indices provide some unification but do not reflect the fundamentally different chemistries operating under acidic, hydrothermal and alkaline pretreatment environments. As a result, reported “optimal conditions” for kapok are often protocol-specific and cannot be readily transferred or interpolated.^18^ An integrated pretreatment–composition landscape showing how cellulose, hemicellulose, and lignin evolve across a common time–temperature–pH-associated pretreatment space is still lacking.

Machine learning (ML) offers a means to extract such integrated relationships from compact but systematically designed data sets.^19^ ML is increasingly being applied in research fields such as chemical materials.^20–22^ Previous studies have discussed lignocellulose for predicting sugar yield, digestibility, and energy parameters, with nonlinear methods capturing interactive effects beyond empirical correlations.^23–25^ However, ML studies rarely target the chemical composition of the pretreated solid itself, and applications to kapok and related hollow fibers are particularly scarce. No prior work has examined dilute acid, hydrothermal, and alkaline conditions within a unified ML framework driven by time, temperature, and pH.

In this study, kapok fiber was used as a model lignocellulosic feedstock to examine how aqueous chemical pretreatment conditions affect cellulose, hemicellulose and lignin contents. Hydrothermal, dilute-acid and alkaline pretreatments were conducted within a shared experimental window, and the resulting data set was used to develop machine-learning models based on residence time, temperature and pH-associated pretreatment environment. Single-output models were first evaluated to determine the predictability of each composition fraction, followed by multioutput models for simultaneous composition prediction. To improve the reliability of model assessment under the limited data set size, retained models were evaluated using LOOCV-based out-of-fold prediction rather than relying only on a single train–test split. Feature-importance analysis was further used to interpret the relative contribution of pretreatment descriptors to the predicted composition responses. This study provides a data-driven assessment of pretreatment–composition relationships in kapok fiber and clarifies the extent to which simple experimental descriptors can support composition prediction after aqueous chemical pretreatment. The overall experimental design and machine-learning framework are summarized in Scheme 1.

**Scheme 1:**
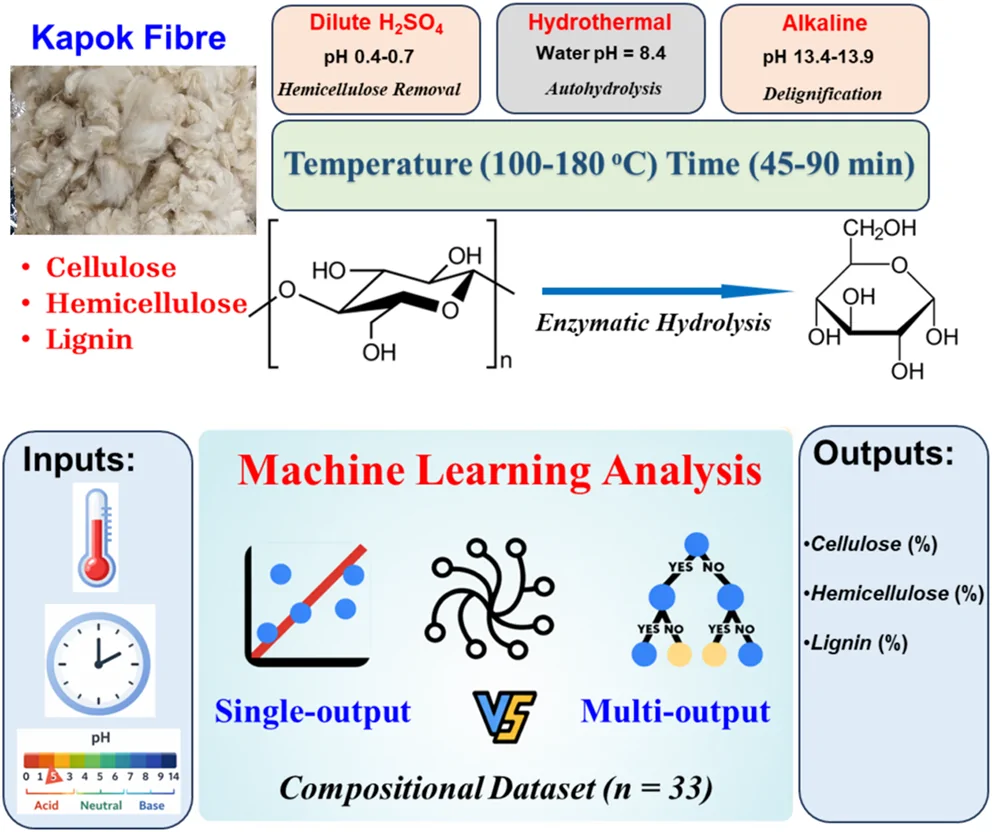
Limited-Data Machine-Learning Concept for Predicting Kapok Fibre Composition after Aqueous Chemical Pretreatment

## 2 Materials and Methods

### 2.1 Biomass Feedstock

Kapok (*Ceiba pentandra*) fiber was sourced from Kelantan, Malaysia. Fibers were manually separated from the seedpods, gently shaken to remove adhering pith and loose debris, and visually cleaned without any chemical purification. A composite batch was prepared by pooling and mixing multiple subsamples to ensure representativeness, after which the material was stored in sealed polyethylene bags at room temperature in the dark. All masses and compositional values reported in this study refer to the dry basis unless otherwise specified.

### 2.2 Chemical Pretreatment

Kapok fibers were subjected to three aqueous chemical pretreatment routes: hydrothermal, dilute-acid and alkaline pretreatment. All pretreatments were conducted in a laboratory-scale stainless-steel digester equipped with microprocessor-controlled heating and an internal thermocouple. The solid-to-liquor ratio was maintained at 1:12.^26^ Hydrothermal pretreatment was conducted with deionized water, whereas dilute-acid and alkaline pretreatments were conducted using 1–2% H_2_SO_4_ and 1–3% NaOH, respectively.^27^ These routes generated three pH-associated pretreatment environments, corresponding to strongly acidic dilute-acid pretreatment, hydrothermal water with a mildly alkaline measured pH, and strongly alkaline pretreatment.

Across the three pretreatment routes, the target temperature ranged from 100 to 180 °C and the residence time ranged from 45 to 90 min. Residence time was counted from the point at which the digester reached the target temperature. After pretreatment, the digester was rapidly cooled, and the slurry was discharged. The pretreated solids were recovered by filtration, washed thoroughly with deionized water until approximately neutral pH, and oven-dried to constant mass before chemical composition analysis.^28^

### 2.3 Chemical Composition Analysis

Untreated and pretreated kapok fibers were air-dried, gently milled and passed through a 2.0 mm sieve prior to analysis. For the untreated material, extractives were removed following TAPPI T 204 (ethanol–toluene), whereas pretreated solids were treated as effectively extractives-free owing to prior liquor contact and subsequent water washing. Acid-insoluble (Klason) lignin was determined according to TAPPI (T222 om-02),^29^ and acid-soluble lignin was quantified spectrophotometrically following TAPPI UM 250.^30^ Holocellulose was determined using the sodium chlorite method, and α-, β- and γ-cellulose fractions were determined using the graded-alkali procedure described in JIS 8101. In this study, cellulose was defined as α-cellulose, hemicellulose was calculated as the sum of β- and γ-cellulose, and lignin was calculated as the sum of acid-insoluble and acid-soluble lignin.

Solid yield was calculated from the oven-dried mass before and after pretreatment according to eq 1. Cellulose and hemicellulose contents in the recovered solid were calculated from their fractions in holocellulose according to eq 2. The final component contents used for modeling were then obtained by combining the measured composition of the recovered solid with the corresponding solid yield according to eq 3. This calculation was applied consistently to cellulose, hemicellulose and lignin. The resulting values therefore represent solid-yield-corrected component contents expressed relative to the initial oven-dried biomass rather than the composition of the recovered solids.

1 $$\mathrm{Solid yield}\;\left(\%\right)=\frac{\mathrm{Final oven‐dried weight of biomass}\;\left(g\right)}{\mathrm{Initial oven‐dried weight of biomass}\;\left(g\right)}\times 100$$

2 Cellulose/Hemicellulose in recovered
solid(%)=[Cellulose/Hemicellulose
fraction in holocellulose(%)×Holocellulose(%)]/100

3 Final component
content(%)=[Component content in recovered
solid(%)×Solid yield(%)]/100

### 2.4 Data Set Construction and Exploratory Analysis

The modeling data set comprised 33 pretreatment runs. The liquor pH used as an input descriptor was measured from the prepared pretreatment liquor before each pretreatment run at room temperature. This pH value therefore represented the initial liquor condition associated with each pretreatment route and was interpreted as a route-associated descriptor rather than as an isolated continuous mechanistic variable. Before modeling, the compiled data set was checked for missing entries, inconsistent units and transcription errors, and all 33 records were retained. The complete data set is provided as Supporting Information Data File S1.

### 2.5 Machine-Learning Modeling and Validation

As shown in Scheme 2, the machine-learning workflow was organized into preliminary algorithm screening followed by LOOCV-based validation of the retained models. In the preliminary screening stage, eight regression algorithms were evaluated for single-output prediction of cellulose, hemicellulose and lignin, including multiple linear regression, ridge regression, decision tree, random forest, gradient boosting decision tree, support vector regression, multilayer perceptron and Gaussian process regression.^31–35^ These algorithms were selected to cover linear baselines, tree-based models, ensemble learners, kernel-based regression, neural-network regression and probabilistic regression. A common 80/20 train–test split was used at this stage only to identify candidate algorithms for each target variable, and the complete screening results are reported in the Supporting Information.

**Scheme 2:**
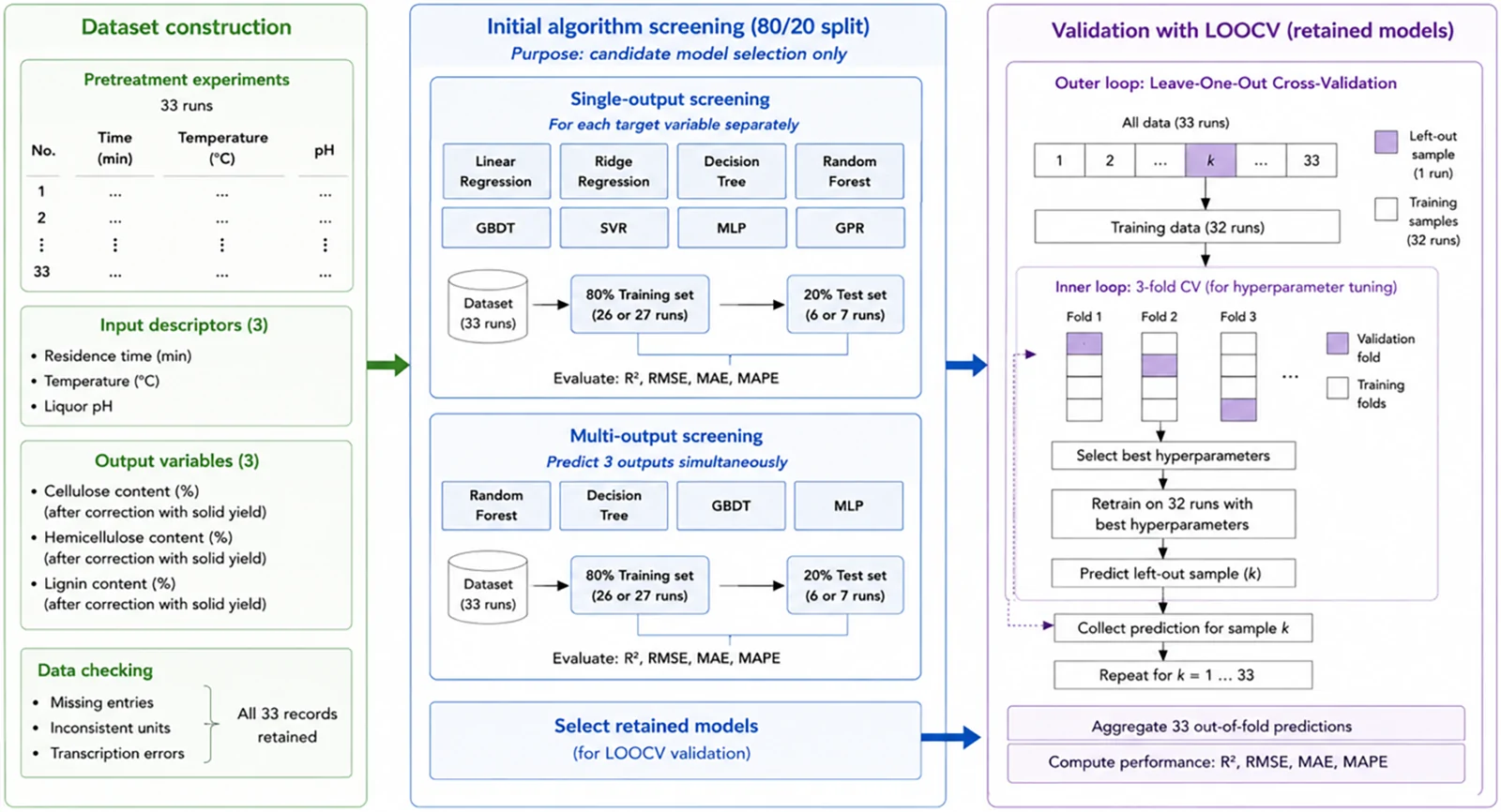
Machine-Learning Workflow for Composition Prediction

The retained single-output models were subsequently evaluated using leave-one-out cross-validation. In each LOOCV iteration, one sample was held out as the validation sample, while the remaining 32 samples were used for model training. For models requiring hyperparameter optimization, parameter tuning was performed within the training portion of each LOOCV iteration using inner 3-fold cross-validation. The held-out sample in the outer LOOCV loop was therefore not used for hyperparameter selection. Final model performance was calculated from the aggregated out-of-fold predictions obtained across all 33 LOOCV iterations.

Wrapper-based multitarget modeling was also conducted to predict cellulose, hemicellulose and lignin simultaneously from the same pretreatment descriptors. Random forest, decision tree, gradient boosting decision tree and multilayer perceptron were evaluated as candidate models within a wrapper-based multitarget regression framework. In this implementation, one target-specific estimator was fitted for each composition output using the same input descriptors and validation procedure. Therefore, the approach was interpreted as simultaneous multitarget prediction rather than as explicit modeling of correlations among cellulose, hemicellulose and lignin. The retained wrapper-based multitarget models were evaluated using the same LOOCV-based validation procedure. Model performance was quantified using the coefficient of determination (*R* ^2^), root mean squared error (RMSE), mean absolute error (MAE) and mean absolute percentage error (MAPE). RMSE and MAE were expressed in percentage points, whereas MAPE was expressed as a percentage.

All machine-learning analyses were performed in Python 3.13.5 using scikit-learn version 1.8.0 for model implementation, cross-validation, hyperparameter tuning and performance evaluation. NumPy version 2.3.5 and pandas version 2.2.3 were used for numerical processing and data handling. The main model classes included LinearRegression, Ridge, DecisionTreeRegressor, RandomForestRegressor, GradientBoostingRegressor, SVR, MLPRegressor and GaussianProcessRegressor. Wrapper-based multitarget RF, DT, GBDT, and MLP models were implemented using MultiOutputRegressor, which fits one estimator per output target. Random states were fixed at 42 for stochastic algorithms where applicable to improve reproducibility. The searched hyperparameter ranges and selected configurations of the retained models are provided in the Supporting Information.

### 2.6 Feature-Importance Analysis

Permutation importance was calculated for the retained models after LOOCV validation. Each input descriptor was randomly permuted independently, and the resulting increase in prediction error was used as its importance value. The permutation procedure was repeated 100 times for each descriptor. RMSE was used as the error function for calculating the increase in prediction error after permutation. For single-output models, permutation importance was calculated separately for cellulose, hemicellulose and lignin. For wrapper-based multitarget models, permutation importance was calculated from the normalized prediction error across the three outputs. The final values were normalized within each model so that the summed importance of residence time, temperature, and pH-associated pretreatment environment equaled one, and were reported as mean ± SD across permutation repeats.

## 3 Results and Discussion

### 3.1 Data Set Overview and Exploratory Composition Patterns

The compiled data set contained 33 pretreatment condition combinations covering residence times of 45–90 min and temperatures of 100–180 °C. The pH values were distributed into three route-associated regimes, corresponding to acid pretreatment with 1–2% H_2_SO_4_ (pH 0.39–0.69), hydrothermal pretreatment (pH ≈ 8.40) and alkaline pretreatment with 1–3% NaOH (pH 13.40–13.88). Across this experimental space, the solid-yield-corrected cellulose, hemicellulose and lignin contents on an initial dry biomass basis ranged from 13.99–48.78%, 5.22–36.94%, and 4.70–18.90%, respectively.

As shown in Figure 1(a), temperature showed the clearest association with the composition variables among the three pretreatment descriptors. Cellulose and hemicellulose were negatively associated with temperature, indicating that stronger thermal exposure was linked to greater polysaccharide loss or redistribution. Time showed the same general direction but with weaker correlations, suggesting that residence time provided less linear explanatory information within the tested range. In contrast, pH showed weak global linear correlations with the individual composition variables when all samples were pooled. This result does not necessarily indicate a negligible chemical effect of pH, but rather reflects that pH in this data set represented route-associated pretreatment environments instead of a continuous independent gradient. Therefore, pH-related effects were expected to appear through route-specific and interaction-dependent responses rather than through a single global linear trend.

**Figure 1:**
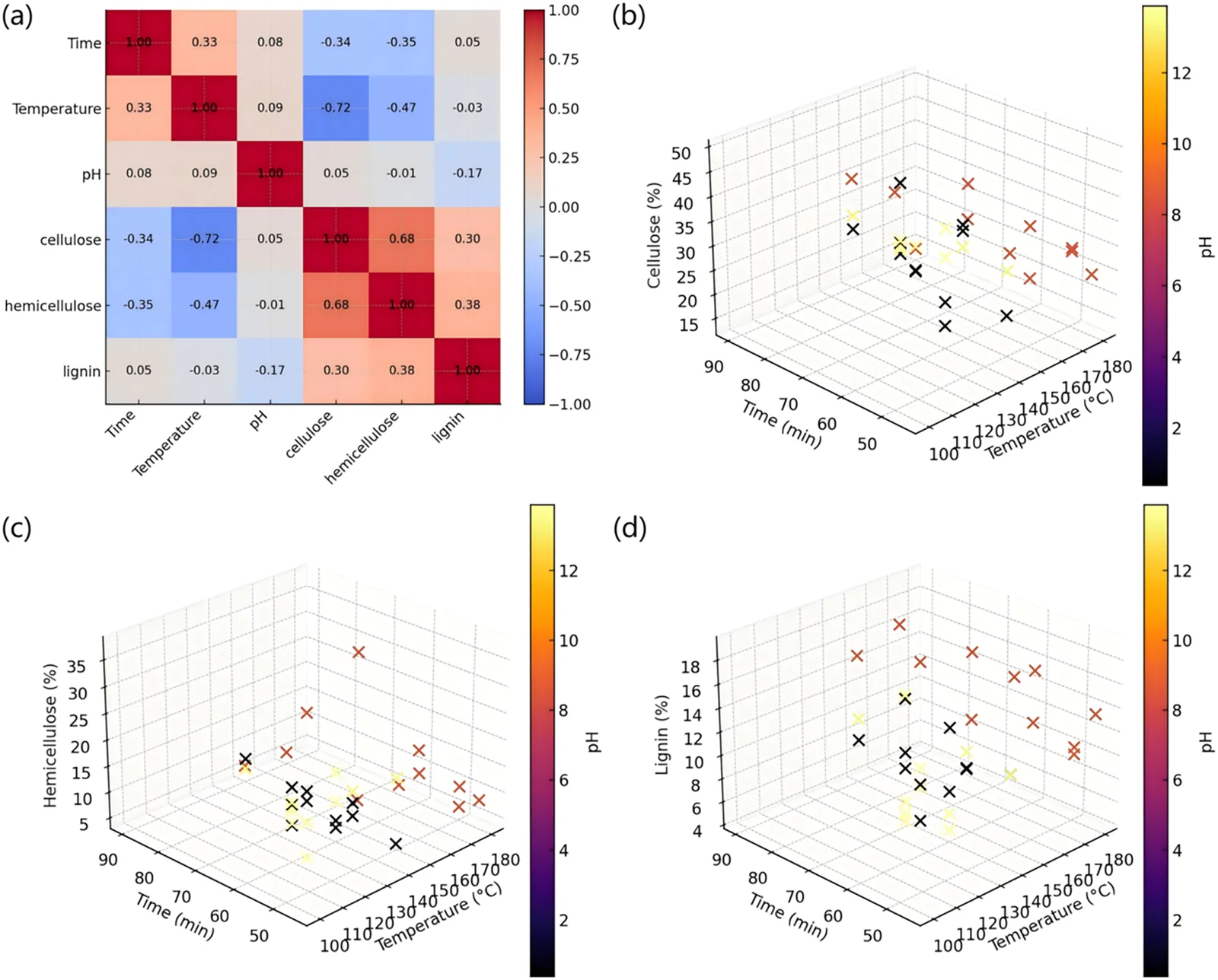
Overview of pretreatment effects on solid-yield-corrected fiber composition: (a) correlations; (b) cellulose; (c) hemicellulose; (d) lignin.

The composition distributions in Figure 1(b–d) further show that cellulose, hemicellulose and lignin responded differently across the time–temperature–pH space. Cellulose-rich samples were mainly observed under moderate thermal conditions, whereas higher temperatures, especially under hydrothermal conditions, were associated with marked cellulose reduction.^36,37^ Hemicellulose showed broader sensitivity to the combined effects of temperature, time and pH-associated pretreatment environment, consistent with its lower thermal stability and greater susceptibility to solubilization during aqueous pretreatment.^38–40^ Lignin varied over a narrower numerical range but displayed a clearer dependence on pretreatment route, with lower lignin contents mainly observed under alkaline conditions at elevated temperature, consistent with alkaline cleavage of lignin–carbohydrate linkages and lignin solubilization.^41,42^ Localized higher lignin values under severe acid or hydrothermal conditions may also reflect lignin condensation, redeposition or pseudolignin formation.^43,44^ These exploratory patterns indicate that the final composition was not governed by a single monotonic severity axis, but by coupled thermal and route-associated chemical effects, supporting the use of machine-learning models to capture nonlinear and interaction-dependent relationships between pretreatment descriptors and fiber composition.

### 3.2 Predictive Performance of Single-Output Machine Learning Models on Fiber Composition

An initial screening was conducted using eight single-output algorithms to identify candidate models for each composition target. The complete train–test screening results are provided in Table S1, and the corresponding graphical comparison is shown in Figure S1. The screening results showed that cellulose was generally more responsive to nonlinear learning than to linear baselines, with GBDT and GPR giving lower test errors than Linear and Ridge models. For hemicellulose, the model ranking was less stable, although MLP gave the strongest test-set performance in the initial screening. For lignin, DT gave the lowest test error among the screened models. Therefore, Ridge, GBDT, and GPR were retained for cellulose, Ridge and MLP were retained for hemicellulose, and DT was retained for lignin. These screened models were then re-evaluated using LOOCV-based out-of-fold prediction to reduce the dependence of performance assessment on a single train–test partition.

After the initial screening, the retained models were further evaluated using LOOCV out-of-fold prediction. This step was used as the main performance assessment because each sample served once as an independent validation point, which reduced the sensitivity of the results to a single train–test split. For models requiring hyperparameter selection, tuning was performed within each LOOCV training fold using inner 3-fold cross-validation, with RMSE as the optimization criterion. The searched hyperparameter ranges and selected configurations are summarized in Table S2, including the fold-wise selection frequencies for GBDT, GPR, Ridge and MLP. The final LOOCV metrics calculated from the out-of-fold predictions are reported in Table 1.

**Table 1:** LOOCV Performance of Selected Target-Specific Single-Output Models^a^

Target	Model	*R* ^2^	RMSE	MAE	MAPE (%)
Cellulose	Ridge	0.427	6.787	5.351	19.09
	GBDT	0.782	4.185	3.446	10.76
	GPR	0.564	5.920	4.603	15.15
Hemicellulose	Ridge	0.122	6.738	5.141	41.46
	MLP	0.026	7.093	5.487	46.73
Lignin	DT	0.216	2.990	2.365	19.88

a Performance metrics were calculated from LOOCV out-of-fold predictions. Where applicable, hyperparameters were selected within each training fold using inner 3-fold grid search with RMSE as the optimization criterion. RMSE and MAE are expressed in percentage points, whereas MAPE is expressed as a percentage.

As shown in Table 1, cellulose showed the strongest LOOCV predictability among the three composition targets. Ridge gave an LOOCV *R* ^2^ of 0.427, with RMSE, MAE and MAPE values of 6.787 percentage points, 5.351 percentage points and 19.09%, respectively, indicating that the linear baseline captured only part of the variation in cellulose content. This result suggests that the relationship between pretreatment conditions and cellulose composition was not fully additive across time, temperature and pH-associated pretreatment environment, which is consistent with the expected nonlinear and interaction-dependent nature of lignocellulosic pretreatment responses.^45,46^ Introducing nonlinear models improved the prediction, with GBDT giving the best cellulose performance, as reflected by an *R* ^2^ of 0.782, RMSE of 4.185 percentage points and MAPE of 10.76%. GPR also improved over Ridge, giving an *R* ^2^ of 0.564 and MAPE of 15.15%, but it remained less accurate than GBDT. Figure 2a shows that GBDT provided closer agreement between measured and LOOCV-predicted cellulose values compared with Ridge and GPR. This improvement indicates that the cellulose response involved nonlinear curvature and variable interactions that the linear baseline could not represent.^47^ Nevertheless, the comparison between the LOOCV results and the stronger apparent performance observed during the initial train–test screening indicates that model flexibility should be interpreted cautiously under the limited and clustered experimental design.^48^

**Figure 2:**
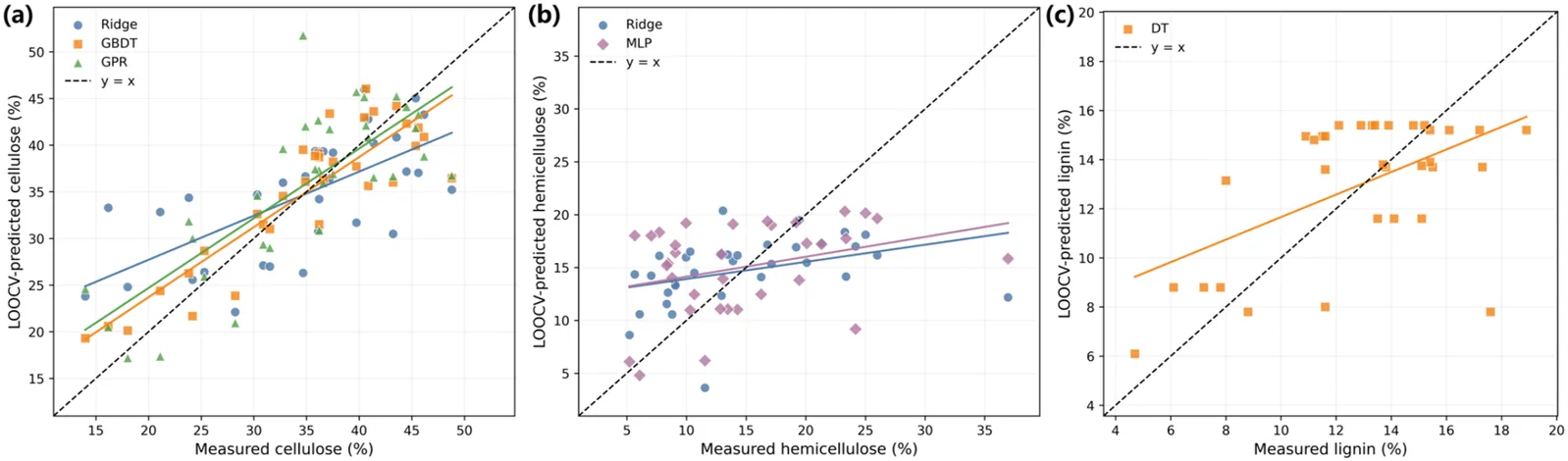
LOOCV-predicted versus measured composition values for selected single-output models: (a) cellulose, (b) hemicellulose, and (c) lignin.

For hemicellulose, the LOOCV results indicated weaker and less stable predictability than cellulose. Ridge gave an *R* ^2^ of 0.122, with RMSE, MAE and MAPE values of 6.738 percentage points, 5.141 percentage points and 41.46%, respectively, showing that the three macroscopic descriptors captured only a limited fraction of the hemicellulose response. MLP did not improve the prediction after LOOCV-based evaluation and hyperparameter tuning, giving a lower *R* ^2^ of 0.026 and a higher MAPE of 46.73%. This result differs from the initial train–test screening, where MLP appeared more favorable, and indicates that its apparent advantage was not retained under the more conservative validation scheme. The scatter distribution in Figure 2(b) also shows a compressed predicted range relative to the measured hemicellulose values, which is consistent with weak target-specific signal recovery from time, temperature and pH-associated pretreatment environment alone. Such limited predictability is reasonable because hemicellulose removal is affected not only by bulk pretreatment conditions but also by localized fiber accessibility, diffusion constraints, xylan substitution patterns and structural heterogeneity that were not explicitly included in the input descriptors.^49,50^

For lignin, the selected DT configuration produced a weaker but still positive LOOCV performance compared with cellulose. The model gave an *R* ^2^ of 0.216, with RMSE, MAE and MAPE values of 2.990 percentage points, 2.365 percentage points and 19.88%, respectively. This result indicates that the selected pretreatment descriptors captured part of the residual lignin variation, but the predictive structure was less reproducible than that observed for cellulose. Figure 2(c) shows that several lignin predictions still deviated substantially from the measured values, suggesting that the DT model mainly recovered broad composition trends rather than precise pointwise lignin responses. This limited predictability is consistent with the complex behavior of lignin during pretreatment, where delignification can occur together with lignin redeposition, pseudolignin formation and condensation reactions, depending on local mass transfer, lignin–carbohydrate associations and liquor chemistry.^51–54^ Therefore, the lignin model should be interpreted as a qualitative indicator of residual-lignin tendency within the explored pretreatment space rather than as a reliable optimization model for lignin content alone.^55^

### 3.3 Wrapper-Based Multitarget Prediction of Fiber Composition

Wrapper-based multitarget modeling was further conducted to predict cellulose, hemicellulose and lignin contents simultaneously from the same pretreatment descriptors. This analysis was used to compare the three composition targets under a consistent modeling and validation framework. Because the wrapper-based implementation fits one estimator per output target, the approach was interpreted as simultaneous multitarget prediction rather than explicit learning of correlations among cellulose, hemicellulose and lignin.

RF, DT, GBDT, and MLP were first evaluated as candidate wrapper-based multitarget models to identify suitable algorithms for simultaneous composition prediction. The complete screening results are provided in Table S3, and the corresponding graphical comparison is shown in Figure S2. Based on the screening results, RF and GBDT were retained for subsequent LOOCV validation. RF was retained as a stable ensemble baseline, whereas GBDT was retained because of its stronger nonlinear learning capacity and favorable prediction performance across the three composition outputs. The hyperparameter settings of the retained models are summarized in Table S4.

Table 2 reports the LOOCV performance of the retained wrapper-based multitarget models. Because cellulose, hemicellulose and lignin differed in content range and response behavior, the prediction metrics were reported separately for each output rather than averaged across outputs. RF produced positive *R* ^2^ values for all three outputs, with the strongest performance observed for cellulose (*R* ^2^ = 0.690; RMSE = 4.994 percentage points; MAPE = 13.47%). Its performance was weaker for hemicellulose and lignin, with *R* ^2^ values of 0.117 and 0.112, respectively, indicating stable but conservative simultaneous prediction. GBDT showed stronger LOOCV performance across the three outputs, with *R* ^2^ values of 0.909, 0.653, and 0.672 for cellulose, hemicellulose and lignin, respectively. The corresponding MAPE values were 6.38%, 21.37% and 11.67%, showing that GBDT provided more accurate wrapper-based simultaneous prediction than RF across the three composition targets.

**Table 2:** LOOCV Performance of Retained Wrapper-Based Multi-Target Models for Simultaneous Composition Prediction^a^

Model	Output	*R* ^2^	RMSE	MAE	MAPE (%)
RF	Cellulose	0.690	4.994	3.808	13.47
	Hemicellulose	0.117	6.756	4.617	35.81
	Lignin	0.112	3.182	2.636	23.22
GBDT	Cellulose	0.909	2.703	2.086	6.38
	Hemicellulose	0.653	4.234	2.611	21.37
	Lignin	0.672	1.934	1.418	11.67

a RF, random forest; GBDT, gradient boosting decision tree; RMSE, root mean squared error; MAE, mean absolute error; MAPE, mean absolute percentage error. RMSE and MAE are expressed in percentage points, whereas MAPE is expressed as a percentage.

Figure 3 provides a visual comparison between the measured and LOOCV-predicted values for the retained wrapper-based multitarget models. For RF, the cellulose predictions showed better agreement with the measured values than the hemicellulose and lignin predictions, consistent with the higher *R* ^2^ and lower MAPE reported for cellulose in Table 2. The hemicellulose predictions showed greater dispersion, indicating that RF only partially captured the variation in hemicellulose content under simultaneous prediction. For GBDT, the predicted values were more closely aligned with the measured values across all three outputs, especially for cellulose and lignin. The reduced dispersion in Figure 3d–f supports the quantitative results in Table 2 and indicates that GBDT provided closer agreement between measured and predicted values under the wrapper-based multitarget framework.

**Figure 3:**
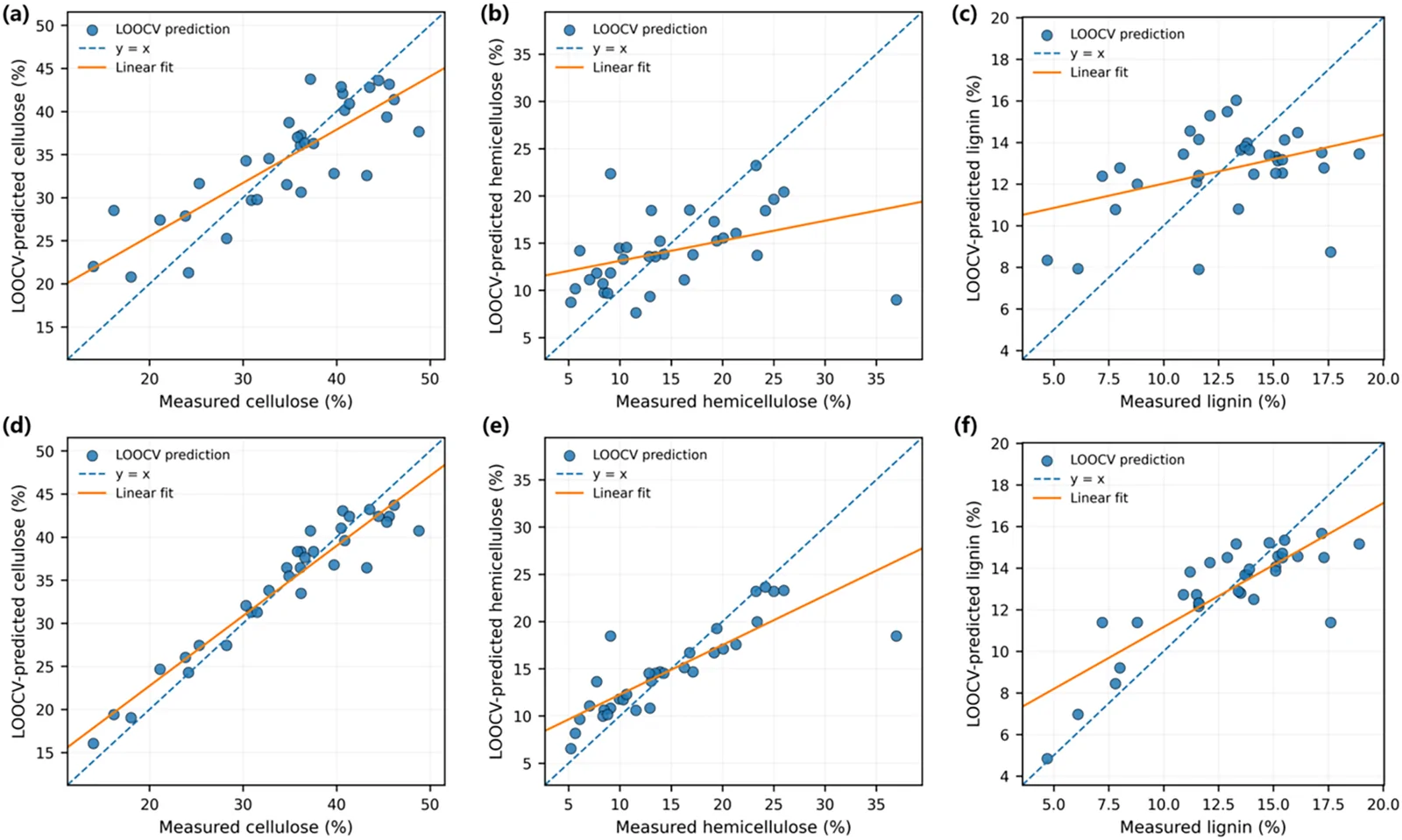
LOOCV-predicted versus measured composition values obtained from the retained wrapper-based multitarget models: (a) RF-cellulose, (b) RF-hemicellulose, (c) RF-lignin, (d) GBDT-cellulose, (e) GBDT-hemicellulose and (f) GBDT-lignin.

The comparison between RF and GBDT indicates that multioutput prediction benefited from boosting-based nonlinear learning. Although RF provided a stable ensemble baseline, its weaker performance for hemicellulose and lignin suggests that bagging-based averaging may smooth part of the target-specific variation in the composition-response patterns. In contrast, GBDT showed stronger prediction across all three outputs, indicating that the boosting-based estimator was more effective for the target-specific nonlinear relationships between pretreatment descriptors and fiber composition. Similar observations have been reported in other data-driven decision-support contexts, where nonlinear intelligent-computing approaches improved the modeling of systems governed by interacting operational variables.^56^ Cellulose remained the most predictable target, whereas hemicellulose was still associated with relatively higher error, reflecting its sensitivity to route-dependent solubilization and structural heterogeneity. The improved lignin prediction obtained by GBDT further suggests that the model better represented residual-lignin patterns within the explored pretreatment space. Therefore, GBDT was selected as the preferred wrapper-based multitarget model for simultaneous composition prediction, while RF was retained as a comparative ensemble baseline.

### 3.4 Feature-Importance Analysis of Pretreatment Descriptors

Feature importance was evaluated using LOOCV-derived permutation importance to interpret how the retained models used time, temperature and pH-associated pretreatment environment for composition prediction. The importance values were normalized within each model so that the three descriptor contributions summed to one, allowing comparison among retained single-output and multioutput models. Because pH values were clustered by pretreatment route, pH-related importance was interpreted as the contribution of the pH-associated pretreatment environment rather than as an isolated continuous pH effect.

As shown in Figure 4(a–c) and Table S5, the retained single-output models showed target-dependent feature-importance patterns. For cellulose, temperature was the dominant descriptor across all retained models, with normalized importance values of 1.000 in Ridge, 0.839 in GBDT and 0.852 in GPR. The pH-associated pretreatment environment showed a secondary contribution in the nonlinear cellulose models, whereas time contributed little. This pattern indicates that cellulose enrichment within the explored pretreatment space was mainly associated with thermal exposure and route-dependent chemical modification.^57^ For hemicellulose, temperature also remained the leading descriptor, with importance values of 0.761 in Ridge and 0.627 in MLP, although time and the pH-associated pretreatment environment contributed more clearly than in cellulose prediction. This broader distribution is consistent with hemicellulose being more sensitive to solubilization, depolymerization and route-specific reactions during aqueous pretreatment.^58,59^ In contrast, lignin prediction showed a different hierarchy, with DT assigning the highest importance to the pH-associated pretreatment environment (0.613), followed by temperature (0.371) and time (0.015). This result agrees with the chemical-route dependence of lignin removal and redistribution during pretreatment, including delignification, condensation and lignin redeposition under different pretreatment environments.^60–62^

**Figure 4:**
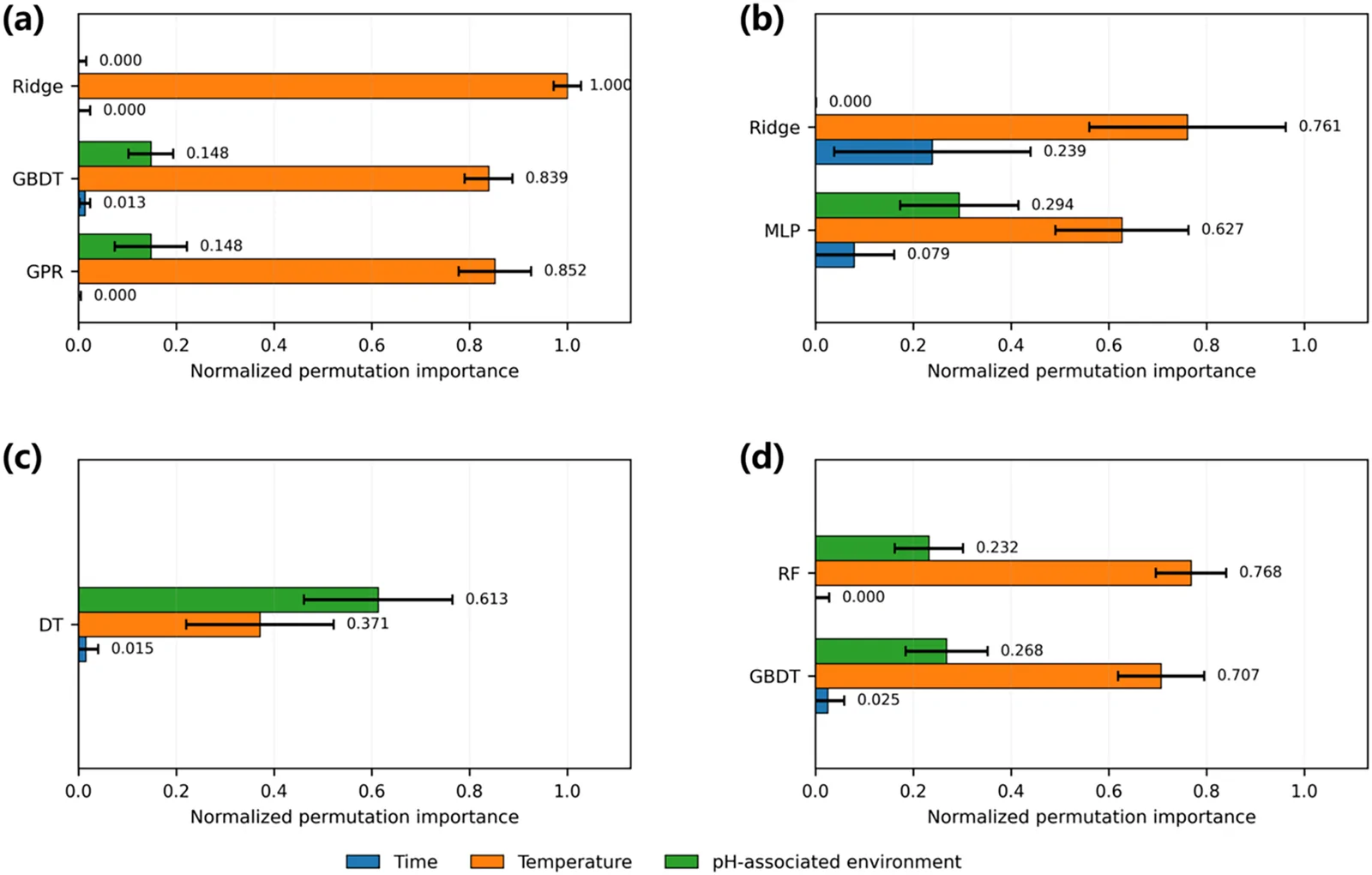
LOOCV-derived normalized permutation importance of time, temperature and pH-associated pretreatment environment in retained models: (a) cellulose single-output models, (b) hemicellulose single-output models, (c) lignin single-output model and (d) retained wrapper-based multitarget models.

The retained multioutput models showed a more integrated feature-importance pattern for simultaneous composition prediction. As shown in Figure 4(d) and Table S5, temperature remained the dominant shared descriptor in both RF and GBDT, with normalized importance values of 0.768 and 0.707, respectively. The pH-associated pretreatment environment was consistently the second most important descriptor, contributing 0.232 in RF and 0.268 in GBDT, whereas time contributed little to the simultaneous prediction. This result indicates that, when cellulose, hemicellulose and lignin were predicted simultaneously under the wrapper-based framework, the models relied mainly on thermal exposure and route-level chemical environment rather than residence time alone. The dominance of temperature in the multioutput models is consistent with the broad influence of thermal severity on biomass fractionation, carbohydrate solubilization and fiber-structure modification during aqueous pretreatment.^63^ Meanwhile, the secondary but consistent contribution of the pH-associated pretreatment environment reflects the route-dependent nature of compositional redistribution, especially for lignin-related changes under acid, hydrothermal and alkaline conditions.

Overall, the feature-importance results indicate that the composition models mainly extracted predictive information from temperature and the pH-associated pretreatment environment, but their relative roles differed among composition targets. Temperature was more influential for cellulose and hemicellulose prediction, suggesting that polysaccharide-related composition changes were primarily associated with thermal exposure and thermally driven fractionation within the investigated pretreatment range. By contrast, the stronger contribution of the pH-associated pretreatment environment to lignin prediction indicates that lignin-related variation was more closely linked to route-dependent chemistry than to residence time alone.^64,65^ The low importance of time should not be interpreted as evidence that residence time is chemically irrelevant, because permutation importance reflects predictive dependence within the current data set rather than an independent causal effect. Within the present experimental design, residence time provided less additional predictive information than temperature and the route-associated chemical environment, probably because the composition responses were more strongly structured by thermal and chemical-route contrasts.

## 4 Conclusion

This study evaluated limited-data machine learning for predicting the cellulose, hemicellulose and lignin contents of kapok fiber after aqueous chemical pretreatment. A data set of 33 pretreatment runs was constructed from hydrothermal, dilute-acid and alkaline pretreatments using residence time, temperature and pH-associated pretreatment environment as input descriptors. To address the limited sample size, model assessment was based on LOOCV out-of-fold prediction after preliminary algorithm screening. The results showed target-dependent predictability. In the single-output models, cellulose was the most predictable component, with GBDT giving the best LOOCV performance, whereas hemicellulose and lignin showed weaker prediction from the same three descriptors. In the wrapper-based multitarget setting, GBDT improved simultaneous prediction across the three composition targets, indicating that target-specific composition responses could be predicted more accurately using boosting-based nonlinear estimators than using the other retained models.

Feature-importance analysis further showed that the models relied mainly on temperature and the pH-associated pretreatment environment, but their relative contributions differed among composition targets. Temperature was the dominant descriptor for cellulose and hemicellulose prediction, supporting the importance of thermal exposure in polysaccharide-related composition changes. By contrast, lignin prediction was more strongly associated with the pH-associated pretreatment environment, reflecting the route-dependent nature of lignin removal, redistribution and secondary reactions under acid, hydrothermal and alkaline conditions. Residence time showed lower importance within the present experimental range, indicating that it provided less additional predictive information than temperature and route-associated chemical environment in this data set.

The main limitation of this study is the compact data set size and the use of only three macroscopic pretreatment descriptors. Therefore, the models should be interpreted as limited-data predictive and exploratory tools rather than as general-purpose optimization models for kapok pretreatment. The clustered pH distribution also means that pH-related importance reflects route-associated pretreatment environment rather than an isolated continuous pH effect. Future work should enlarge the experimental matrix, include independent external validation, and incorporate additional descriptors such as solid yield, liquor composition, severity-related indices, fiber morphology, crystallinity and lignin-structure information. Linking these enriched descriptors to downstream responses, such as enzymatic digestibility, sugar release or material performance, would strengthen the use of data-driven modeling for sustainable kapok fiber valorization.

## Supplementary Material





## Data Availability

The data set supporting the findings of this study, is provided as a Supporting Information data file.

## Notes

Raw data (XLSX)
Initial screening results for single-output and wrapper-based multitarget models; hyperparameter search ranges and selected model configurations; prediction-performance figures; permutation-importance results; complete data set, solid-yield values, model inputs and modeling targets (PDF)

## Abbreviations

CV: cross-validation
DT: decision tree
GBDT: gradient boosting decision tree
GPR: Gaussian process regression
LOOCV: leave-one-out cross-validation
MAE: mean absolute error
MAPE: mean absolute percentage error
ML: machine learning
MLP: multilayer perceptron regressor
RF: random forest
RMSE: root mean squared error
R^2^: coefficient of determination
SD: standard deviation
SVR: support vector regression

## References

[1] S J., K A., Kamalakannan A., S S., Arumugam A., Muthuraman M. S. (2025). Advancements
and the Prospects of Biodegradable Films Fabricated with Biological
Macromolecules for Sustainable Food Packaging through Scientometric
Analysis: A Review. ACS Food Sci. Technol..

[2] Aguilar G. J., De Oliveira A. M., Augusto P. E. D., Tapia-Blácido D. R. (2025). Advances
in Eco-Friendly Materials for Sustainable Packaging and Single-Use
Utensils: A Decade of Innovation in Preparation, Characterization,
and Applications. ACS Appl. Mater. Interfaces.

[3] Tye Y. Y., Lee K. T., Wan Abdullah W. N., Leh C. P. (2013). Potential of *Ceiba
pentandra* (L.) Gaertn. (Kapok) Fiber as a Resource
for Second Generation Bioethanol: Parametric Optimization and Comparative
Study of Various Pretreatments Prior Enzymatic Saccharification for
Sugar Production. Bioresour. Technol..

[4] Mo W., Ke K., Shen X., Li B. (2020). The Influence of “Thermal
Drying Pretreatment” on Enzymatic Hydrolysis of Cellulose and
Xylan in Poplar Fibers with High Lignin Content. Carbohydr. Polym..

[5] Purnawati R., Febrianto F., J Wistara I. N., Nikmatin S., Hidayat W., Lee S. H., Kim N. H. (2018). Physical
and Chemical Properties
of Kapok (Ceiba Pentandra) and Balsa (Ochroma Pyramidale) Fibers. J. Korean Wood Sci. Technol..

[6] Madadi M., Kargaran E., Al Azad S., Saleknezhad M., Zhang E., Sun F. (2025). Machine Learning-Driven
Optimization
of Biphasic Pretreatment Conditions for Enhanced Lignocellulosic Biomass
Fractionation. Energy.

[7] Niu Y., Hemashini T., Leh C. P. (2026). Comparative Energy Assessment and
Net Energy Return Analysis of Non-Wood Lignocellulosic Bioethanol
Systems. Cellulose.

[8] Ying T. Y., Teong L. K., Abdullah W. N. W., Peng L. C. (2014). The Effect of Various
Pretreatment Methods on Oil Palm Empty Fruit Bunch (EFB) and Kenaf
Core Fibers for Sugar Production. Procedia Environ.
Sci..

[9] Oladzad S., Fallah N., Mahboubi A., Afsham N., Taherzadeh M. J., Toghyani J. (2024). Comparison of Acid and Hydrothermal Pretreatments of
Date Waste for Value Creation. Sci. Rep..

[10] Lu Y., He Q., Fan G., Cheng Q., Song G. (2021). Extraction and Modification
of Hemicellulose from Lignocellulosic Biomass: A Review. Green Process. Synth..

[11] Yang J., Gao C., Yang X., Su Y., Shi S., Han L. (2022). Effect of
Combined Wet Alkaline Mechanical Pretreatment on Enzymatic Hydrolysis
of Corn Stover and Its Mechanism. Biotechnol.
Biofuels Bioprod..

[12] Wang W., Wang X., Zhang Y., Yu Q., Tan X., Zhuang X., Yuan Z. (2020). Effect of Sodium Hydroxide Pretreatment
on Physicochemical Changes and Enzymatic Hydrolysis of Herbaceous
and Woody Lignocelluloses. Ind. Crops Prod..

[13] Shibly M. A. H., Arman Md. H., Sabur Md. A., Bhuiyan M. A. H. (2024). Agro-Waste from *Gladiolus hybrida* Plants: Effects of Alkaline Processing
on a New Natural Cellulosic Fiber Derived for Polymer Composites. ACS Sustainable Resour. Manage..

[14] Dong T., Xu G., Wang F. (2015). Adsorption
and Adhesiveness of Kapok Fiber to Different
Oils. J. Hazard. Mater..

[15] Cao Y., Xie L., Sun G., Su F., Kong Q.-Q., Li F., Ma W., Shi J., Jiang D., Lu C., Chen C.-M. (2018). Hollow
Carbon Microtubes from Kapok Fiber: Structural Evolution and Energy
Storage Performance. Sustainable Energy Fuels.

[16] Singh C. J., Mukhopadhyay S., Rengasamy R. S., Srivastava M., Kumari R. (2025). Comparative Analysis
of *Calotropis procera* and *Ceiba pentandra* Fibre-Based Filters
Used to Separate Oil from Emulsified Effluent. RSC Sustainability.

[17] Chan E. W. C., Yeong S. W., Wong C. W., Soo O. Y. M., Phua A. C. Y., Ng Y. K. (2022). Ceiba Pentandra
(L.) Gaertn.: An Overview of Its Botany,
Uses, Reproductive Biology, Pharmacological Properties, and Industrial
Potentials. J. App Biol. Biotech.

[18] Scapini T., dos Santos M. S. N., Bonatto C., Wancura J. H. C., Mulinari J., Camargo A. F., Klanovicz N., Zabot G. L., Tres M. V., Fongaro G., Treichel H. (2021). Hydrothermal Pretreatment of Lignocellulosic
Biomass for Hemicellulose Recovery. Bioresour.
Technol..

[19] Azad S. A., Madadi M., Rahman A., Sun C., Sun F. (2025). Machine Learning-Driven
Optimization of Pretreatment and Enzymatic Hydrolysis of Sugarcane
Bagasse: Analytical Insights for Industrial Scale-Up. Fuel.

[20] Raza A., Hu Y., Lu Y. (2024). Improving Carbon Flux Estimation in Tea Plantation
Ecosystems: A Machine Learning Ensemble Approach. Eur. J. Agron..

[21] Dong C., Zhu H., Wang J., Yuan H., Zhao J., Chen Q. (2017). Prediction
of Black Tea Fermentation Quality Indices Using NIRS and Nonlinear
Tools. Food Sci. Biotechnol..

[22] Han F., Huang X., Teye E. (2019). Novel Prediction
of Heavy Metal Residues
in Fish Using a Low-Cost Optical Electronic Tongue System Based on
Colorimetric Sensors Array. J. Food Process
Eng..

[23] Niu, Y. ; Lee, C. K. ; Leh, C. P. Kapok Pod Fibre as a Sustainable Biofuel Resource: Prediction of Cellulose Hydrolysis Using a Heuristic Algorithm Optimized Random Forest. In Proceedings of the 4th International Symposium in Environmental Science and Industrial Ecology; Zhu, Y. , Ed.; Springer Nature: Singapore, 2025; pp 205–214 10.1007/978-981-96-1578-0_15.

[24] Niu Y., Tye Y. Y., Hemashin T., Leh C. P. (2025). Feasibility of Use
Limited Data to Establish a Relationship between Chemical Composition
and the Enzymatic Glucose Yield Using Machine Learning. Biomass Bioenergy.

[25] Niu Y., Tye Y. Y., Lee C. K., Ahmad M. I., Leh C. P. (2025). Composition-Centered
Prediction of Kenaf Core Saccharification for next-Generation Bioethanol
via Machine Learning. Front. Fuels.

[26] Niu Y., Tye Y. Y., Lee C. K., Leh C. P. (2026). Composition Retention
Metrics Reveal Route-Specific Controls of Glucose Release from Pretreated
Kenaf Core. ACS Omega.

[27] Niu Y., Tye Y. Y., Lee C. K., Leh C. P. (2026). Chemical Composition
Retention Perspective: Research on Pretreatment Production of Lignocellulose
Bioethanol. Biofuels.

[28] Niu Y., Wang Y., Joseph N., Abiodun A. K., Leh C. P., Lee C. K. (2026). Time-Resolved Co-Fermentation Dynamics: Optimizing
Fermentation Time for Energy-Efficient Lignocellulosic Ethanol Production
from Oil Palm Empty Fruit Bunches. Energy Sustainable
Dev..

[29] Tappi, T. Acid-Insoluble Lignin in Wood and Pulp, TAPPI Test Methods; 2006.

[30] Ehrman, C. I. Methods for the Chemical Analysis of Biomass Process Streams. In Handbook on Bioethanol; Routledge, 1996.

[31] Arifuzzaman M., Hasan M. R., Toma T. J., Hassan S. B., Paul A. K. (2023). An Advanced
Decision Tree-Based Deep Neural Network in Nonlinear Data Classification. Technologies.

[32] Adler A. I., Painsky A. (2022). Feature Importance in Gradient Boosting
Trees with
Cross-Validation Feature Selection. Entropy.

[33] Fawagreh K., Gaber M. M., Elyan E. (2014). Random Forests:
From Early Developments
to Recent Advancements. Syst. Sci. Control Eng..

[34] Awad, M. ; Khanna, R. Support Vector Regression. In Efficient Learning Machines: Theories, Concepts, and Applications for Engineers and System Designers; Awad, M. ; Khanna, R. , Eds.; Apress: Berkeley, CA, 2015; pp 67–80 10.1007/978-1-4302-5990-9_4.

[35] Zhang, F. ; O’Donnell, L. J. Support Vector Regression. In Machine Learning; Mechelli, A. ; Vieira, S. , Eds.; Academic Press, 2020, Chapter 7; pp 123–140 10.1016/B978-0-12-815739-8.00007-9.

[36] Knill C. J., Kennedy J. F. (2003). Degradation of Cellulose
under Alkaline Conditions. Carbohydr. Polym..

[37] Li Q., Wang A., Ding W., Zhang Y. (2016). Influencing Factors
for Alkaline Degradation of Cellulose. BioResources.

[38] Ruiz H. A., Galbe M., Garrote G., Ramirez-Gutierrez D. M., Ximenes E., Sun S.-N., Lachos-Perez D., Rodríguez-Jasso R. M., Sun R.-C., Yang B., Ladisch M. R. (2021). Severity Factor Kinetic Model as a Strategic Parameter
of Hydrothermal Processing (Steam Explosion and Liquid Hot Water)
for Biomass Fractionation under Biorefinery Concept. Bioresour. Technol..

[39] Gallina G., Alfageme E. R., Biasi P., García-Serna J. (2018). Hydrothermal
Extraction of Hemicellulose: From Lab to Pilot Scale. Bioresour. Technol..

[40] Gautam D., Rana V., Sharma S., Walia Y. K., Kumar K., Umar A., Ibrahim A. A., Baskoutas S. (2025). Hemicelluloses:
A Review on Extraction and Modification for Various Applications. ChemistrySelect.

[41] Trajano H. L., Engle N. L., Foston M., Ragauskas A. J., Tschaplinski T. J., Wyman C. E. (2013). The Fate of Lignin
during Hydrothermal
Pretreatment. Biotechnol. Biofuels.

[42] Jung S., Trajano H. L., Yoo C. G., Foston M. B., Hu F., Tolbert A. K., Wyman C. E., Ragauskas A. J. (2018). Topochemical
Understanding of Lignin Distribution During Hydrothermal Flowthrough
Pretreatment. ChemistrySelect.

[43] Hu F., Jung S., Ragauskas A. (2012). Pseudo-Lignin Formation and Its Impact
on Enzymatic Hydrolysis. Bioresour. Technol..

[44] Wu R., Wang X., Zhang Y., Fu Y., Qin M. (2022). Efficient
Removal of Surface-Deposited Pseudo-Lignin and Lignin Droplets by
Isothermal Phase Separation during Hydrolysis. Bioresour. Technol..

[45] Kim Y., Kreke T., Mosier N. S., Ladisch M. R. (2014). Severity Factor
Coefficients for Subcritical Liquid Hot Water Pretreatment of Hardwood
Chips. Biotechnol. Bioeng..

[46] Batista G., Souza R. B. A., Pratto B., dos Santos-Rocha M. S. R., Cruz A. J. G. (2019). Effect of Severity
Factor on the Hydrothermal Pretreatment
of Sugarcane Straw. Bioresour. Technol..

[47] Namboonlue S., Ngowsakul K., Nakarat K., Kongsinkaew C., Subjalearndee N., Uttayopas P., Charoenrat T., Laemthong T. (2025). Predictive
Reducing Sugar Release from Lignocellulosic
Biomass Using Sequential Acid Pretreatment and Enzymatic Hydrolysis
by Harnessing a Machine Learning Approach. Comput.
Struct. Biotechnol. J..

[48] Gholizadeh M., Jamei M., Ahmadianfar I., Pourrajab R. (2020). Prediction
of Nanofluids Viscosity Using Random Forest (RF) Approach. Chemom. Intell. Lab. Syst..

[49] Li J., Liu Y., Duan C., Zhang H., Ni Y. (2015). Mechanical Pretreatment
Improving Hemicelluloses Removal from Cellulosic Fibers during Cold
Caustic Extraction. Bioresour. Technol..

[50] Xie T., Fan M. (2025). Machine Learning Models
for Predicting Enzymatic Hydrolysis Yields
of Lignocellulosic Biomass after Various Pretreatments. Ind. Crops Prod..

[51] Feng N., She S., Tang F., Zhao X., Chen J., Wang P., Wu Q., Rojas O. J. (2023). Formation
and Identification of Lignin–Carbohydrate
Complexes in Pre-Hydrolysis Liquors. Biomacromolecules.

[52] Ji Q., Yu X., Wu P., El-Gasim A Yagoub A., Chen L., Mustapha A. T., Zhou C. (2021). Pretreatment of Sugarcane Bagasse with Deep Eutectic Solvents Affect
the Structure and Morphology of Lignin. Ind.
Crop. Prod..

[53] Ji Q., Yu X., El-Gasim
A Yagoub A., Chen L., Zhou C. (2020). Efficient
Removal of Lignin from Vegetable Wastes by Ultrasonic and Microwave-Assisted
Treatment with Ternary Deep Eutectic Solvent. Ind. Crops Prod..

[54] Li H., Liang J., Chen L., Ren M., Zhou C. (2023). Utilization
of Walnut Shell by Deep Eutectic Solvents: Enzymatic Digestion of
Cellulose and Preparation of Lignin Nanoparticles. Ind. Crops Prod..

[55] Baruah J., Nath B. K., Sharma R., Kumar S., Deka R. C., Baruah D. C., Kalita E. (2018). Recent Trends in the
Pretreatment
of Lignocellulosic Biomass for Value-Added Products. Front. Energy Res..

[56] Zhang Y., Yuqi Z., Liang Y., Mu S., Zhang Z., Chen X. (2025). AD-VGF: An Improved Generative Adversarial
Network for Credit Card
Risk Identification and Management in Digital Economy. Cybern. Syst..

[57] Yu S., Dong X., Zhao P., Luo Z., Sun Z., Yang X., Li Q., Wang L., Zhang Y., Zhou H. (2022). Decoupled Temperature and Pressure Hydrothermal Synthesis of Carbon
Sub-Micron Spheres from Cellulose. Nat. Commun..

[58] Mosier N., Wyman C., Dale B., Elander R., Lee Y. Y., Holtzapple M., Ladisch M. (2005). Features of Promising Technologies
for Pretreatment of Lignocellulosic Biomass. Bioresour. Technol..

[59] Ghosh D., Tanner J., Lavoie J.-M., Garnier G., Patti A. F. (2021). An Integrated
Approach for Hemicellulose Extraction from Forest Residue. BioResources.

[60] Tarasov D., Leitch M., Fatehi P. (2018). Lignin–Carbohydrate
Complexes:
Properties, Applications, Analyses, and Methods of Extraction: A Review. Biotechnol. Biofuels.

[61] Wan G., Zhang Q., Li M., Jia Z., Guo C., Luo B., Wang S., Min D. (2019). How Pseudo-Lignin
Is Generated during
Dilute Sulfuric Acid Pretreatment. J. Agric.
Food Chem..

[62] Shinde S. D., Meng X., Kumar R., Ragauskas A. J. (2018). Recent
Advances in Understanding the Pseudo-Lignin Formation in a Lignocellulosic
Biorefinery. Green Chem..

[63] Wang, J. ; Deng, J. ; Rashwan, T. L. ; Qu, G. ; Ren, S. ; Liu, D. Oxygen-Lean Thermokinetics and Early-Warning of Laojunmiao Non-Caking Coal in Xinjiang Coalfield Int. J. Min. Sci. Technol. 2026 10.1016/j.ijmst.2026.07.005.

[64] Zhao Y., Shang Z., Yan T., Zhong H., Wang H., Wang Y., Chen W., Jiao W., Li J., Zhou Y., Liu J., Qu G. (2026). Atomic Editing of the
First-Shell to Modulate the d-Electron State of Transition Metal Sites
for Accelerated Redox Kinetics in Lithium–Sulfur Batteries. Adv. Mater..

[65] Da G., Wang C., Peng R. (2027). Reliability
of Wireless Sensor Networks
with Probabilistic Functional Dependence: A Lifetime-Based Approach. Reliab. Eng. Syst. Safety.

