# Supplementary material for: Small Sample Machine Learning for Predicting Composition Changes in Chemically Pretreated Kapok Fiber
Source: ACS Omega. 2026 Sep 23;11(39):59120–30. doi: 10.1021/acsomega.6c04765 (PMC13647415; doi:10.1021/acsomega.6c04765)
Supplement: Supplementary file 1 [file ao6c04765_si_002.pdf]

Small Sample Machine Learning for Predicting Composition Changes in Chemically Pretreated Kapok Fibre

Yitong Niu[†], Ireland LaBass[‡], Ying Ying Tye[†], Tristan Smith[‡], Sicheng Wang[‡],

Yunxiang Li[‡], Ting Han[‡], Cheu Peng Leh[†]**

[†]School of Industrial Technology, Universiti Sains Malaysia, 11800 Gelugor, Penang, Malaysia

[‡]Division of Chemistry and Biochemistry, Texas Woman's University, Denton, TX 76204, United States

Supplementary Tables

Table S1. Performance of machine-learning models for predicting cellulose, hemicellulose and lignin contents.

Model	Train					Test				
	**MSE**	**RMSE**	**MAE**	**MAPE (%)**	**R^2**	**MSE**	**RMSE**	**MAE**	**MAPE (%)**	**R^2**
**Cellulose**										
Linear	29.518	5.433	4.5	15.3	0.618	63.292	7.956	5.824	22.51	0.309
Ridge	29.518	5.433	4.5	15.3	0.618	63.287	7.955	5.823	22.51	0.309
DT	1.056	1.028	0.394	1	0.986	39.205	6.261	4.864	14.19	0.572
RF	3.887	1.971	1.654	5.67	0.95	44.635	6.681	4.976	16.88	0.512
GBDT	1.094	1.046	0.523	1.46	0.986	38.448	6.201	4.694	13.8	0.58
SVR	62.1	7.881	5.84	23.28	0.195	81.35	9.019	6.474	26.33	0.111
MLP	26.05	5.104	4.299	14.38	0.662	64.44	8.027	6.597	22.63	0.296
GPR	2.465	1.57	1.136	3.93	0.968	37.619	6.133	4.508	13.68	0.589
**Hemicellulose**										
Linear	41.411	6.435	4.6	34.93	0.287	25.481	5.048	4.267	43.677	0.082
Ridge	41.411	6.435	4.6	34.93	0.287	25.481	5.048	4.267	43.677	0.082
DT	15.089	3.884	1.221	8.298	0.74	21.441	4.63	4.269	31.547	0.228
RF	18.542	4.306	2.438	21.286	0.681	25.045	5.005	4.708	36.393	0.098
GBDT	15.283	3.909	1.428	10.284	0.737	28.249	5.315	4.95	35.334	0
SVR	50.284	7.091	4.99	35.863	0.134	22.768	4.772	4.096	37.175	0.18
MLP	26.919	5.188	3.503	26.645	0.536	15.09	3.885	3.205	25.45	0.457
GPR	39.311	6.27	4.931	40.687	0.323	22.177	4.709	4.205	39.888	0.201
**Lignin**										
Linear	11.398	3.376	2.713	26.925	0.077	11.056	3.325	2.755	24.305	0
Ridge	11.398	3.376	2.713	26.925	0.077	11.049	3.324	2.753	24.295	0
DT	3.016	1.737	0.712	5.992	0.756	4.693	2.166	1.636	13.808	0.401
RF	3.743	1.935	1.353	12.072	0.697	9.306	3.051	2.486	20.955	0
GBDT	3.06	1.749	0.857	7.138	0.752	7.488	2.736	2.155	18.425	0.044
SVR	9.775	3.126	2.327	24.948	0.209	7.987	2.826	2.236	20.394	0
MLP	4.431	2.105	1.547	12.739	0.641	13.388	3.659	3.291	28.391	0
GPR	6.662	2.581	2.097	20.133	0.461	7.713	2.777	2.125	18.727	0.016

Notes: DT: decision tree; RF: random forest; GBDT: gradient boosting decision tree; SVR: support vector regression; MLP: multilayer perceptron regressor; GPR: Gaussian process regression; MSE: mean squared error; RMSE: root mean squared error; MAE: mean absolute error; MAPE: mean absolute percentage error.

Table S2. Hyperparameter search ranges and selected configurations for LOOCV-based single-output models**.**

Target	Model	Hyperparameters searched	Selected configuration	Times selected
Cellulose	Ridge	α = 0.0001, 0.001, 0.01, 0.1, 1, 10, 100, 1000, 10000	α = 1	—
	GBDT	n_estimators = 50, 100; learning_rate = 0.05, 0.10; max_depth = 1, 2; min_samples_leaf = 1, 3	n_estimators = 100; learning_rate = 0.10; max_depth = 2; min_samples_leaf = 1	17
			n_estimators = 100; learning_rate = 0.05; max_depth = 2; min_samples_leaf = 1	8
			n_estimators = 50; learning_rate = 0.10; max_depth = 2; min_samples_leaf = 1	8
	GPR	kernel = RBF, Matern; length_scale = 0.5, 1.0, 2.0; α = 0.001, 0.01, 0.1; Matern ν = 1.5	RBF; length_scale = 2.0; α = 0.001	20
			Matern; length_scale = 2.0; α = 0.001; ν = 1.5	8
			RBF; length_scale = 2.0; α = 0.01	5
Hemicellulose	Ridge	α = 0.0001, 0.001, 0.01, 0.1, 1, 10, 100, 1000, 10000	α = 10	31
			α = 1	2
	MLP	hidden_layer_sizes = (5,); activation = tanh, relu; α = 0.01, 0.1; learning_rate_init = 0.001	hidden_layer_sizes = (5,); activation = relu; α = 0.10; learning_rate_init = 0.001	20
			hidden_layer_sizes = (5,); activation = relu; α = 0.01; learning_rate_init = 0.001	13
Lignin	DT	criterion = absolute_error; max_depth = 3; min_samples_leaf = 1	criterion = absolute_error; max_depth = 3; min_samples_leaf = 1	—

Notes: Times selected indicates the number of outer LOOCV folds in which the configuration was selected by the inner tuning procedure. RMSE was used as the inner tuning objective where fold-wise tuning was applied.

Table S3. Performance of multi-output machine learning models for predicting cellulose, hemicellulose and lignin contents.

Model	Output	Train					Test				
		MSE	RMSE	MAE	MAPE (%)	R^2	MSE	RMSE	MAE	MAPE (%)	R^2
RF	Cellulose	4.586	2.142	1.706	5.994	0.941	48.783	6.986	5.059	17.220	0.467
	Hemicellulose	18.363	4.285	2.431	21.003	0.684	18.243	4.271	4.053	32.449	0.343
	Lignin	3.631	1.905	1.333	11.829	0.706	7.677	2.771	2.259	19.248	0.020
DT	Cellulose	1.056	1.028	0.394	1.010	0.986	38.868	6.237	4.820	14.090	0.575
	Hemicellulose	15.089	3.884	1.221	8.321	0.740	21.441	4.630	4.269	31.551	0.228
	Lignin	3.016	1.737	0.712	5.992	0.756	14.003	3.742	3.050	26.170	0.000
GBDT	Cellulose	1.094	1.046	0.523	1.462	0.986	38.448	6.200	4.694	13.793	0.580
	Hemicellulose	15.283	3.909	1.428	10.279	0.737	28.249	5.315	4.950	35.329	0.000
	Lignin	3.060	1.749	0.857	7.138	0.752	7.488	2.736	2.155	18.425	0.044
MLP	Cellulose	30.172	5.493	4.517	14.986	0.778	45.358	6.733	5.818	19.292	0.278
	Hemicellulose	39.560	6.290	4.300	32.834	0.435	21.984	4.689	4.001	38.845	0.208
	Lignin	9.845	3.138	2.498	21.716	0.407	10.230	3.198	2.555	23.347	0.000

Notes: DT: decision tree; RF: random forest; GBDT: gradient boosting decision tree; MLP: multilayer perceptron regressor; MSE: mean squared error; RMSE: root mean squared error; MAE: mean absolute error; MAPE: mean absolute percentage error.

Table S4. Hyperparameter settings of the retained multi-output models.

Model	Hyperparameters searched	Selected	Selection frequency
RF	n_estimators: 20, 50; max_depth: 2, 3, None; min_samples_leaf: 1, 3; random_state: 42	n_estimators = 50; max_depth = None; min_samples_leaf = 1; random_state = 42	23/33
		n_estimators = 20; max_depth = 3; min_samples_leaf = 1; random_state = 42	4/33
		n_estimators = 20; max_depth = None; min_samples_leaf = 1; random_state = 42	4/33
		n_estimators = 50; max_depth = 3; min_samples_leaf = 1; random_state = 42	2/33
GBDT	n_estimators: 20, 50; learning_rate: 0.05, 0.10; max_depth: 1; min_samples_leaf: 1; random_state: 42	n_estimators = 50; learning_rate = 0.10; max_depth = 1; min_samples_leaf = 1; random_state = 42	19/33
		n_estimators = 20; learning_rate = 0.10; max_depth = 1; min_samples_leaf = 1; random_state = 42	11/33
		n_estimators = 20; learning_rate = 0.05; max_depth = 1; min_samples_leaf = 1; random_state = 42	3/33

Notes: RF denotes random forest and GBDT denotes gradient boosting decision tree. Hyperparameter selection was conducted within each outer LOOCV training fold using inner 3-fold cross-validation.

Table S5. LOOCV-derived permutation importance of pretreatment descriptors in retained single-output and multi-output models.

Model type	Target	Model	Time	Temperature	pH-associated pretreatment environment
Single-output	Cellulose	Ridge	0.000 ± 0.024	1.000 ± 0.028	0.000 ± 0.016
	Cellulose	GBDT	0.013 ± 0.011	0.839 ± 0.049	0.148 ± 0.046
	Cellulose	GPR	0.000 ± 0.005	0.852 ± 0.074	0.148 ± 0.074
	Hemicellulose	Ridge	0.239 ± 0.201	0.761 ± 0.201	0.000 ± 0.001
	Hemicellulose	MLP	0.079 ± 0.082	0.627 ± 0.136	0.294 ± 0.121
	Lignin	DT	0.015 ± 0.025	0.371 ± 0.151	0.613 ± 0.152
Multi-output	Simultaneous composition	RF	0.000 ± 0.028	0.768 ± 0.072	0.232 ± 0.070
	Simultaneous composition	GBDT	0.025 ± 0.034	0.707 ± 0.088	0.268 ± 0.084

Notes: Values are normalized LOOCV-derived permutation importance values and are reported as mean ± SD. For each model, the mean importance values of time, temperature and pH-associated pretreatment environment sum to 1. Multi-output importance was calculated using normalized prediction error across cellulose, hemicellulose and lignin to reduce scale effects among the three composition outputs. RF, random forest; DT, decision tree; GBDT, gradient boosting decision tree; GPR, Gaussian process regression; MLP, multilayer perceptron.

Supplementary Figures

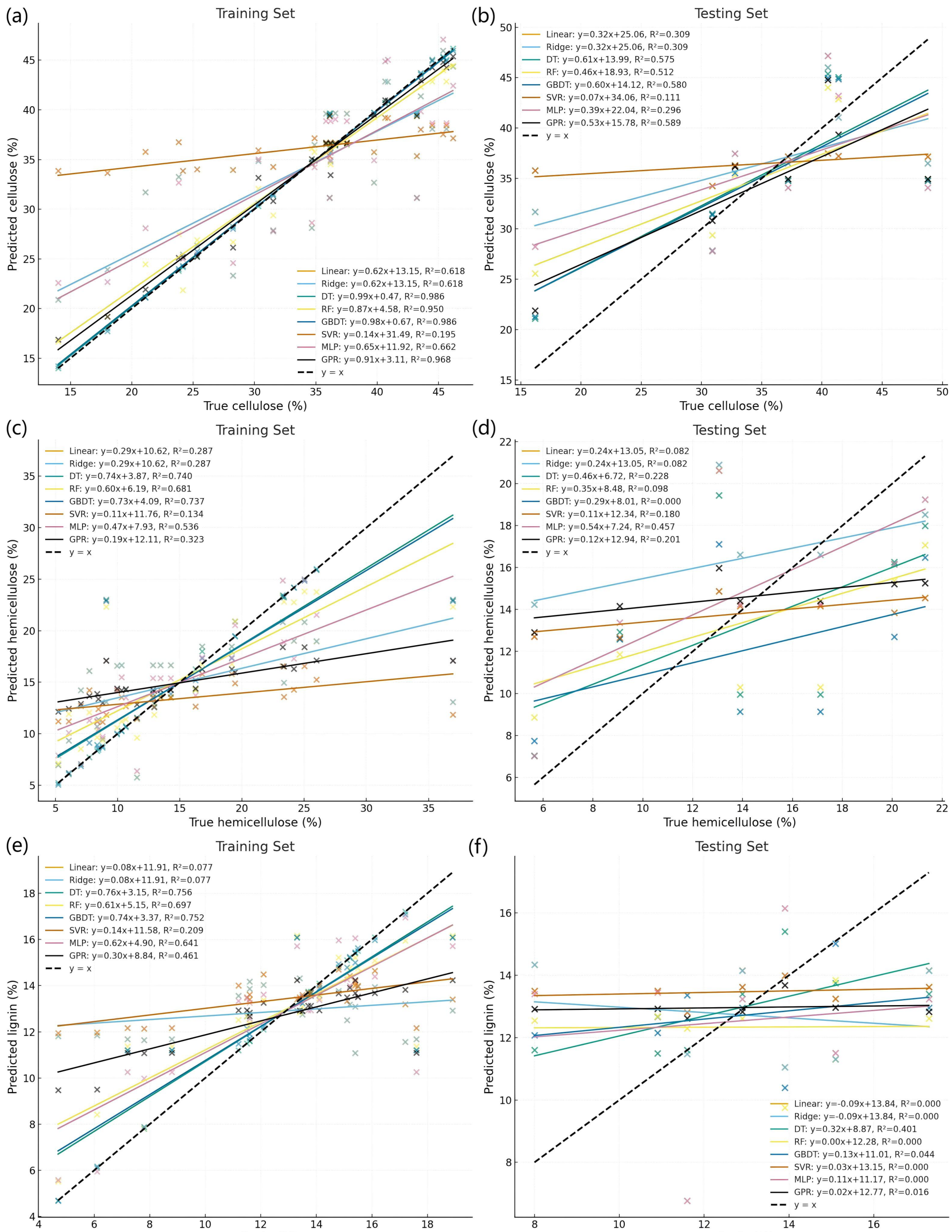

Figure S1. Initial screening performance of single-output models for cellulose, hemicellulose and lignin prediction: (a) cellulose, training set; (b) cellulose, testing set; (c) hemicellulose, training set; (d) hemicellulose, testing set; (e) lignin, training set; and (f) lignin, testing set.

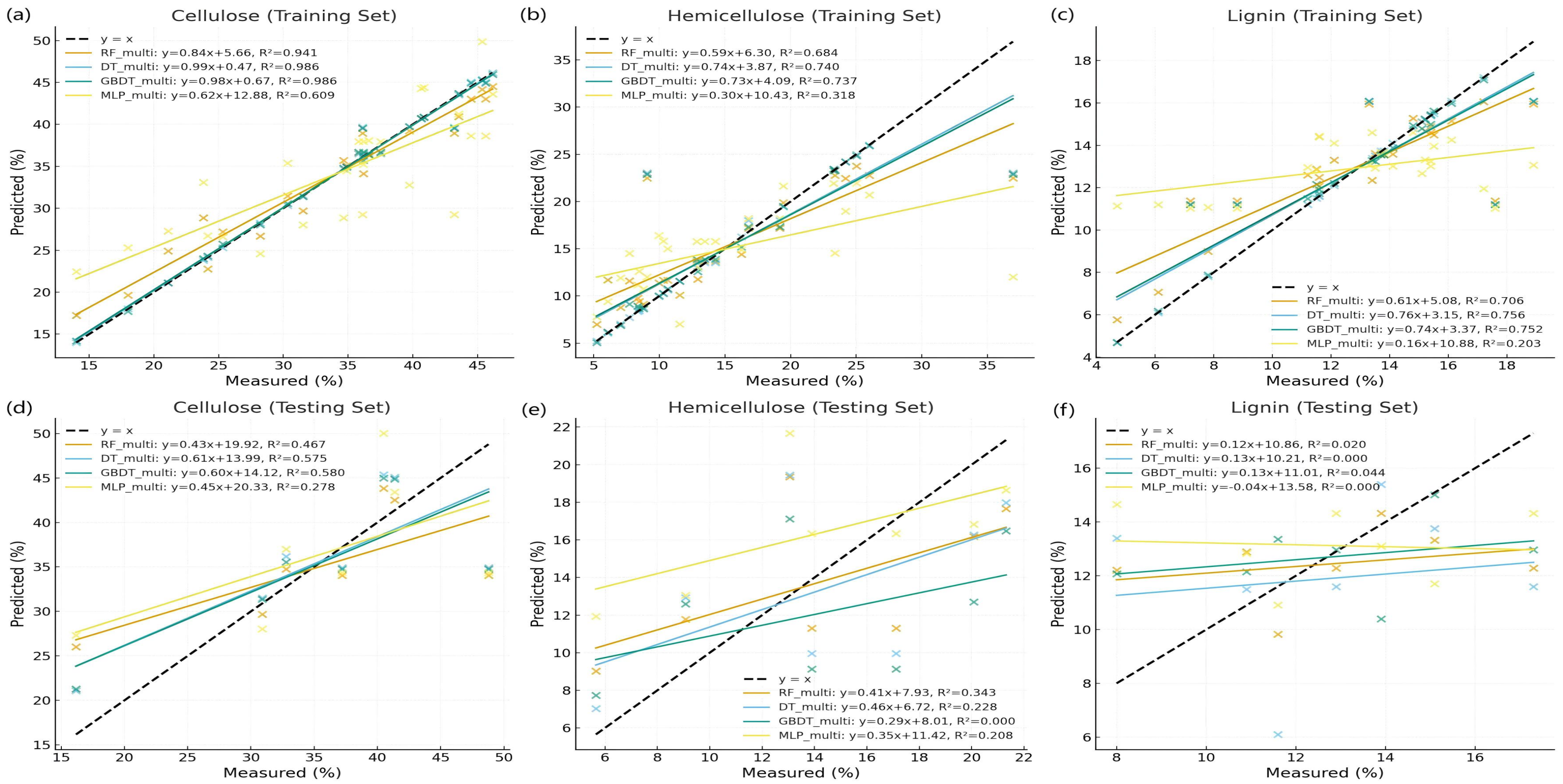

Figure S2. Initial screening performance of wrapper-based multi-target models for simultaneous cellulose, hemicellulose and lignin prediction: (a) cellulose, training set; (b) hemicellulose, training set; (c) lignin, training set; (d) cellulose, testing set; (e) hemicellulose, testing set; and (f) lignin, testing set.
